# Depression among wives of migrant workers in Shuklagandaki Municipality, Tanahun District: A cross-sectional study in Nepal

**DOI:** 10.1371/journal.pmen.0000484

**Published:** 2025-10-30

**Authors:** Anjana Sigdel, Ishwori Byanju Shrestha, Richa Aryal, Manish Rajbanshi, Nand Ram Gahatraj

**Affiliations:** 1 School of Health and Allied Sciences, Pokhara University, Kaski, Nepal; 2 Department of Psychology, Padma Kanya Multiple Campus, Tribhuvan University, Kathmandu, Nepal; 3 Central Department of Public Health, Institute of Medicine, Tribhuvan University, Kathmandu, Nepal; PLOS: Public Library of Science, UNITED KINGDOM OF GREAT BRITAIN AND NORTHERN IRELAND

## Abstract

International migration offers opportunities for personal, social, and economic growth for migrant workers, but results in household and childcare responsibilities among left-behind wives in their home country. While handling all those responsibilities alone, these women feel isolated and may become victims of depression. This study aimed to assess the prevalence of depression and factors associated with depression among wives of migrant workers. A cross-sectional study was conducted among 255 wives of migrant workers in Shuklagandaki municipality, Tanahun. A multistage sampling method was adopted, and face-to-face interviews were administered for data collection. The Center for Epidemiologic Studies Depression Scale (CES-D) was used to assess the prevalence of depression. Chi-square and bivariate logistic regression were performed for bivariate analysis. Multivariate logistic regression analysis determined the factors associated with the prevalence of depression. All the tests were performed at a 95% Confidence Interval, and variables with a p-value < 0.05 were considered statistically significant. The mean ± SD age of the participants was 33.4 ± 6.9 years. The majority belonged to the Janajati ethnicity (44.7%) and had agriculture as their main occupation (49%). Nearly one-fourth (23.5%) of the wives of migrant workers had a prevalence of depression. Family type (AOR: 1.65, 95% CI: 1.21–2.01), family debt (AOR: 2.23, 95% CI: 1.19–5.89), alcohol consumption (AOR: 1.76, 95% CI: 1.15–5.56) and emotional family support (AOR: 1.51, 95% CI: 1.12–1.96) were statistically significant with the prevalence of depression. Nearly one-fourth of wives of migrant workers experienced depression, influenced by ethnicity, family type, marriage type, family debt, and illness. Targeted interventions, including community mental health programs, family counselling, financial support initiatives, routine health screenings, and peer networks, alongside prioritization in national mental health policies, are crucial to improve their well-being.

## Introduction

Depression is a leading contributor to the overall global burden of disease, which is characterized by persistent low mood or diminished interest in activities for an extended period [1]. International Migration offers opportunities for personal, social, and economic growth for migrant workers, but it also poses challenges, such as exploitation and violence for their families, particularly wives left behind in the home country [2].

Approximately 322 million people across the globe are suffering from depression, around half of them live in South-East Asia and the Western-Pacific Region [3]. Studies from Bangladesh and Sri Lanka had reported the depression prevalence of 28.36% [4] and 12.3% [5] respectively, among spouses of migrant workers. Epidemiological studies have identified that women experience depressive disorder more than two times as often as men in their lifespans [6]. More than 80% of the burden of depression is observed in low-and middle-income countries (LMIC) such as Nepal [3].

In Nepal, a national representative assessment found that the prevalence of depression among people aged 18–65 was 11.7% [7]. The migration of Nepali workers abroad has increased every year [8]. The majority (81.28%) of migrant workers from Nepal were men as per the National Census, 2021 [8]. This phenomenon has a positive impact on socio-economic conditions but affects families’ mental health. Nepalese studies indicate that the prevalence of depression among the wives of migrant workers ranges widely, from as low as 6.6% [9] to as high as 79% [10]. Among these, mild depression (27%) and moderate depression (8.5%) were prominent among women [11].

Several studies showed that depression was significantly higher among wives of migrant workers compared to wives of non-migrant workers [5,12,13]. Previous studies have highlighted that depression is influenced by various factors such as family structure, financial debt, autonomy, alcohol use, and family support, which collectively shape mental health outcomes [7,14–16]. As for left-behind wives, the migration of their husbands is one of the main stressors causing psychological issues such as stress, depression, etc. [17].

Women’s autonomy is often limited by patriarchal household structures, typically led by male heads in Nepal [18]. Women who assume the role of de facto household heads in the absence of their husbands often carry greater responsibilities and a heavier workload, but gain increased autonomy and decision-making power [19]. In traditional and conservative families, wives left-behind who live with in-laws frequently face stricter control and reduced freedom, which may increase psychological stress and vulnerability to depression [10,19]. These variations in family structure and household dynamics can significantly influence the mental well-being of left-behind wives.

Despite the high prevalence of depression, very few studies have been conducted regarding depression among wives of migrant workers in Nepal [9–11]. Research on depression among wives of migrant workers is important because this group faces unique stressors such as prolonged separation, increased household responsibilities, financial challenges, and limited social support, which heighten their vulnerability to mental health problems. Their well-being directly affects family functioning and children’s development, yet their psychological needs are often overlooked. Therefore, this study aimed to assess the prevalence and factors associated with depression among the wives of migrant workers in Shuklagandaki Municipality, Tanahun. Evidence generated through this research is essential to guide targeted mental health interventions, strengthen community support mechanisms, and shape responsive policies, while also contributing to the limited global knowledge on the mental health impacts of migration on left-behind spouses.

## Materials and methodology

### Study design and settings

A cross-sectional study was conducted among wives of migrant workers residing in selected wards of Shuklagandaki Municipality, Tanahun [20]. It is located in Gandaki Province, which comprises a total of 12 wards. This municipality has a total of 29,730 female population, out of which reproductive age women of 15–49 years were 17,121 [21]. This site was selected as one of the high migrant population originating areas.

### Study population

The study participants were women aged 15–49 whose husbands had worked abroad for at least six months. Participants who have been residing for at least 6 months were included. Women with hearing disabilities and speech disorders were excluded from the study.

### Sample size and sampling techniques

A sample size (n = 255) was determined by using the Cochran proportionate formula (Z^^2^^pq/e^^2^^), assuming a 95% Confidence Interval (CI), 5% margin of error (e), and an estimated prevalence of depression (p) of 0.79 from a similar study [10]. A multistage sampling technique was used to select the participants. Seven wards were randomly selected among 12 wards in the municipality. Then, four toles per ward were chosen using a simple random sampling method. Four selected toles of corresponding wards consisted of a total of 363 (Ward A = 51, Ward B = 52, Ward C = 59, Ward D = 51, Ward E = 53, Ward F = 44, Ward G = 53) married females whose husbands were abroad, identified through the household survey. An equal proportionate sampling technique was followed to select participants from the 4 selected toles. Then, the required number of households were selected randomly from each tole with the help of community people.

### Data collection procedure

Face-to-face interviews were carried out using structured questionnaires after obtaining the participants’ written consent. The Principal Investigator (AS) was involved in the data collection. The data collection was conducted from 20 January to 22 February 2023. Each interview took around 30 minutes to complete. Female Community Healthcare Volunteers were responsible for identifying participants’ houses.

### Tools and measures

Depression was measured using a validated tool, the Center for Epidemiologic Studies Depression Scale (CES-D) [22]. The CES-D is a 20-item standardized tool on a 4-point Likert scale ranging from 0 (rarely or none of the time) to 3 (most or all the time). Higher scores indicate a greater likelihood of depression. Participants’ responses were scored (Rarely = score 0, Sometimes = score 1, Occasionally = score 2, Most of the time = score 3). Items 4,8, 12, and 16 were reverse scored. Validation of the Nepali version demonstrated good internal consistency (Cronbach’s α = 0.82) [23] and identified a cut-off score of 16 as an appropriate threshold for detecting sub-threshold depression [22]. Furthermore, the questionnaire was pretested on 10% of the sample (n = 26) in a nearby municipality to check the internal consistency of the tool.

A study tool consisted of four sections. The first section included socio-economic and demographic information (age, ethnicity, education, marriage type, duration of marriage, family type, having children, number of children, main occupation, residency, own named property, main source of income, average monthly income, and family debt).

The second section included behavior and health-related information (smoking/tobacco use, alcohol consumption, physical activity and its type, foods, and snacks consumed frequently, and health illness and their types).

The third section included autonomy and migration-related information (household head, decision regarding husband’s earnings, decision regarding healthcare, decision regarding purchase of goods, family and social support, involvement in community groups, occupation of husbands, and remittance received). The last section included depression-related information such as restlessness, loneliness, poor appetite, tearfulness, etc.

### Data management and analysis

Data were systematically entered, cleaned, coded, and verified using Epi-Data version 3.1, while analysis was conducted with SPSS version 25.0 (IBM). Descriptive statistics, including frequencies, percentages, means, and standard deviations, were used to present individual characteristics. Associations between individual characteristics and depression were initially examined using Chi-square tests and bivariate binary logistic regression. The variables with cell count per event less than 10 were excluded from the multivariate regression model. A multicollinearity test was performed among the variables before regression analysis. Multiple regression models were developed with different variable inclusion thresholds (p < 0.1, 0.2, and 0.3) and compared with a full model informed by theoretical relevance. The final model included variables with p < 0.2, which were entered into a multivariable logistic regression to adjust for potential confounders. Model fit was assessed using the Hosmer–Lemeshow test and the Nagelkerke R-squared value. Statistical significance was set at p < 0.05, and adjusted odds ratios (AOR) with 95% confidence intervals (CI) were reported to reflect the strength and precision of associations.

### Ethical approval

The study was reviewed and approved by the Institutional Review Committee of Pokhara University (Ref: 73–079/80). A letter of support was taken from the municipality office. Both verbal and written consent were obtained from each participant after explaining the purpose of the study. The assent form was taken from the participants under 18 years old. The parental consent form was obtained for the participants under 18 years old. The participant’s information was kept confidential, and autonomy was maintained throughout the study. Participants showing depressive symptoms were counseled and referred to local health facilities.

## Results

### Socio-economic and demographical characteristics of the participants

A total of 255 women participated in this study. The mean (±SD) age of the participants was 33.4 ± 6.9 years. Most of the participants belonged to the Janajati ethnicity (44.7%), attained secondary level education (59.6%), had arranged marriage (67.8%), and were homemakers (78.8%). Additionally, more than half (54.1%) of the participants didn’t have property in their name. (Table 1)

**Table 1:** Socio-economic and demographical characteristics of the participants (n = 255).

Characteristics	Number (n)	Percentage (%)
**Age (in years)**		
** Mean±SD, Range**	33.4 ± 6.9,15-49	
** **15-29	74	29.0
** **30-39	135	52.9
** **40-49	46	18.1
**Ethnicity**		
** **Brahmin/Chhetri	82	32.2
** **Janajati	114	44.7
** **Dalit	59	23.1
**Education level**		
** **Informal	5	2.0
** **Primary level	79	31.0
** **Secondary level	152	59.6
** **Bachelor level or above	19	7.4
**Marriage type**		
** **Arrange	173	67.8
** **Love	57	22.4
** **Eloped	25	9.8
**Duration of marriage (in years)**		
**Median (IQR), Range**	14 (9-19), 1-32	
** ** ≤ 5	32	12.5
** **6-10	46	18.0
** **11-15	67	26.3
** **More than 15	110	43.2
**Family type**		
** **Nuclear	161	63.1
** **Joint/Extended	94	36.9
**Having children**		
** **Yes	242	94.9
** **No	13	5.1
**Number of children(n = 242)**		
** **Less than 3	204	84.3
** **3 and more	38	15.7
**Main occupation**		
** **Agriculture	125	49.0
** **Homemaker	79	31.0
** **Business	29	11.4
** **Service/Job	7	2.7
** **Others (labour, tailor, wages)	15	5.9
**Own named property**		
** **Yes	117	45.9
** **No	138	54.1
**Average monthly income (In NRs)**		
** **Median (IQR), Range	50,000 (40,000-70,000), 10,000-4,00,000	
** ** < NRs 50,000 (USD = 356.05)	154	60.4
** **NRs 50,000–100,000 (USD = 356.05-712.10)	93	36.5
** ** > NRs 100,000 (USD 712.10)	8	3.1
**Family debt**		
** **No	104	40.8
** **Yes	151	59.2

### Behavioral and health-related characteristics of the participants

In this study, tobacco and alcohol consumption were low. All participants consumed rice, lentils, and vegetables for lunch/dinner, whereas most consumed junk foods in snacks such as noodles (84.3%) and biscuits (80%). Similarly, less than a fifth (15.7%) of the participants had suffered from health illness within the last six months, among which common illnesses were hypertension (27.5%), gastritis (17.5%), and mental illness (15%). (Table 2)

**Table 2:** Behavioral and health-related characteristics of the participants.

Characteristics	Number (n)	Percentage (%)
**Smoking/tobacco use**		
Yes	2	0.8
No	253	99.2
**Alcohol consumption**		
Yes	25	9.8
No	230	90.2
**If yes, how often**		
Special Occasion	25	100
**Physical activity**		
Yes	255	100
**Type of physical activity**		
Light intensity	243	95.3
Moderate intensity	12	4.7
Foods consumed frequently*		
Rice	255	100
Lentil	255	100
Green vegetables	255	100
Roti	3	1.2
Meat	3	1.2
Snacks consumed frequently*		
Tea	218	85.5
Noodles	215	84.3
Biscuit	204	80.0
Roti/Bread	194	76.1
Beaten/Puffed rice	188	73.7
Chowmein	60	23.5
Curry	34	13.3
Mo: mo	15	5.9
Samosa	10	3.9
**Health illness within the last 6 months**		
Yes	40	15.7
No	215	84.3
Type of health illness (n = 40) *		
Hypertension	11	27.5
Gastritis	7	17.5
Typhoid	6	15.0
Mental illness	6	15.0
Diabetes	5	12.5
Heart disease	3	7.5
Allergy	2	5.0
Asthma	2	5.0
Covid 19	2	5.0
Severe Diarrhea	2	5.0
Others (Thyroidism, Uterus problem, Pneumonia etc.)	9	22.5

*Multiple response questions

### Autonomy and migration-related characteristics of the participants

Table 3 shows that the majority of the participants themselves were household heads (80.4%), decision-makers regarding health care (85.9%), and purchase of goods (87.1%). Two-fifths (21.6%) of the migrated husbands of participants were laborers (21.6%) and drivers (20.4%). Most participants reported that their husbands used to send remittances monthly (77.6%).

**Table 3:** Autonomy and migration-related characteristics of the participants.

Characteristics	Number (n)	Percentage (%)
**Household head**		
Self	205	80.4
Mother-in-law	31	12.2
Father-in-law	16	6.3
Husband	1	0.4
Both (husband and self)	1	0.4
Other (brother-in-law)	1	0.4
**Decision regarding husband’s earning**		
Both (husband and self)	164	64.3
Self	53	20.8
Mother-in-law	20	7.8
Father-in-law	14	5.5
Husband	3	1.2
Other (brother-in-law)	1	0.4
**Decision regarding healthcare**		
Self	219	85.9
Mother-in-law	20	7.8
Father-in-law	11	4.3
Both (husband and self)	5	2.0
**Decision regarding purchase of goods**		
Self	222	87.1
Mother-in-law	21	8.2
Father-in-law	11	4.3
Other (brother-in-law)	1	0.4
**Support of family in decision**		
Yes	252	98.8
No	3	1.2
**Physical Support (n = 252)**		
Yes	197	78.2
No	55	21.8
**Emotional Support (n = 252)**		
Yes	231	91.7
No	21	8.3
**Social support received**		
Yes	248	97.3
No	7	2.7
**Physical Social support received (n = 248)**		
Yes	27	10.9
No	221	89.1
**Emotional Social support received (n = 248)**		
Yes	156	62.9
No	92	37.1
**Involvement in community groups**		
Yes	153	60
No	102	40
**Occupation of husband**		
Laborer	55	21.6
Driver	52	20.4
Hotel worker	40	15.7
Security guard	34	13.3
Technician	13	5.1
Agricultural worker	6	2.4
Others	55	21.6
**Frequency of remittance received**		
Monthly	198	77.6
2–3 months	51	20.0
Semi yearly	3	1.2
Yearly	3	1.2
**Communication with husband**		
Daily	247	96.9
Often	6	2.4
Sometimes	2	0.8
**Main medium to communicate**		
Facebook/Messenger	111	43.5
Imo	109	42.8
Botim	22	8.6
WhatsApp	10	3.9
Totalk	3	1.2

### Prevalence of Depression

**Fig 1:**
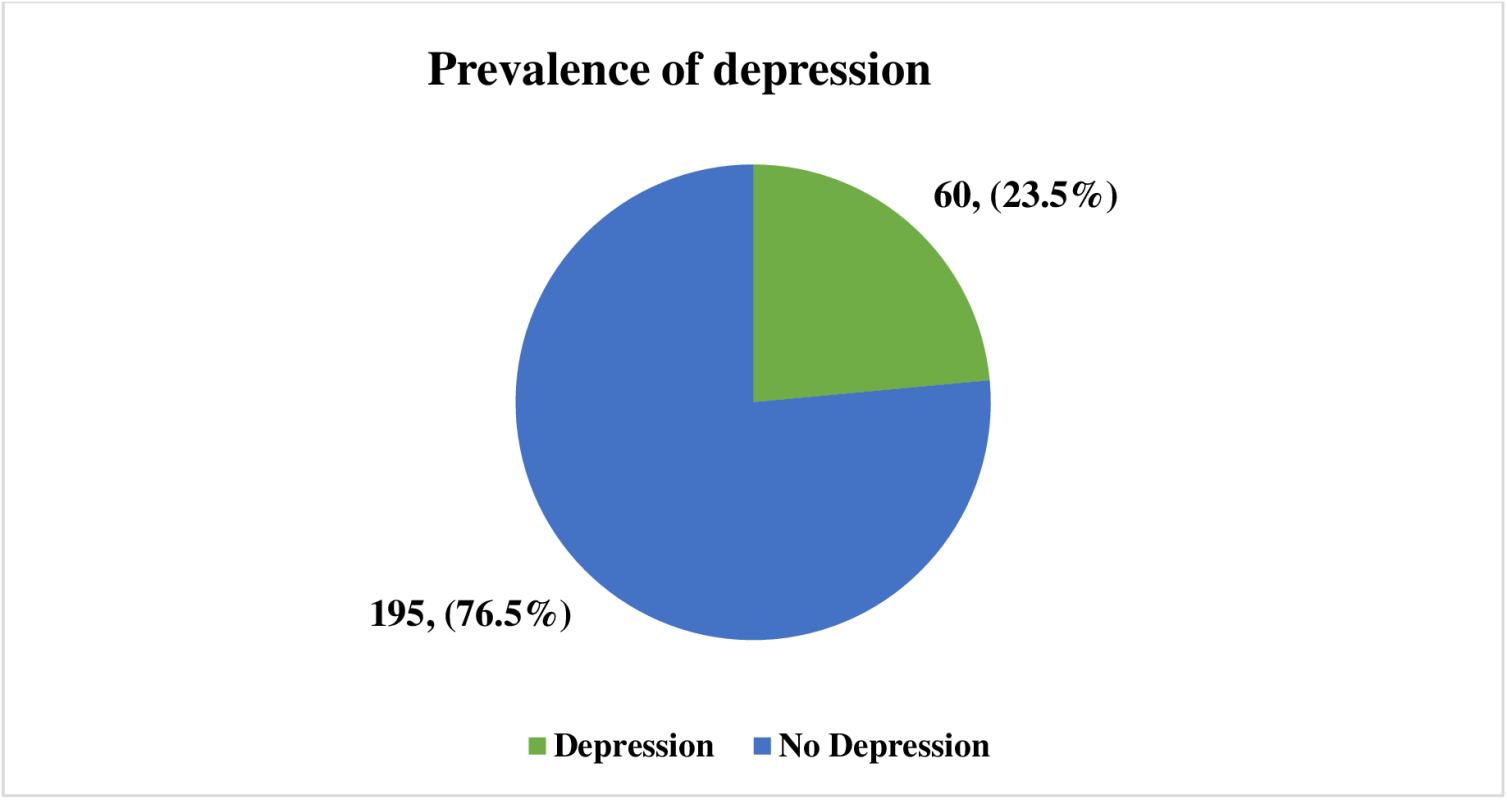
Prevalence of depression.

The study revealed that 23.5% of participants were found to have depression, while the remaining 76.5% did not exhibit depressive symptoms. (Fig 1)

### Association between socio-economic demographical characteristics and depression

Table 4 depicts higher odds of depression among the participants who belonged to a nuclear family (AOR: 1.65, 95% CI: 1.21–2.01, p = 0.020) than other family types. Similarly, participants having family debt (AOR: 2.23, 95% CI: 1.19–5.89, p = 0.002) were two times more likely to have depression compared to those who did not have family debt.

**Table 4:** Multivariate logistic regression analysis between socio-economic demographics and depression, (n = 255).

Characteristics	Depression		Bivariate analysis	Multivariate logistic regression analysis
	Yes	No	COR (95% CI)	AOR (95% CI)
	n (%)	n (%)		
**Age (in years)**				
15-29	15 (20.3)	59 (79.7)	Ref	
30-39	33 (24.4)	102 (75.6)	0.78 (0.39-1.56)	
40-49	12 (26.1)	34 (73.9)	0.72 (0.30-1.71)	
**Ethnicity**				
Janajati	26 (22.8)	88 (77.2)	Ref	Ref
Brahmin/Chettri	11 (13.4)	71 (86.6)	1.90 (0.88-4.12)	1.39 (0.55- 3.49)
Dalit	23 (39.0)	36 (61.0)	0.40 (0.23-0.91)	0.50 (0.21-1.16)
**Education level**				
Secondary and above	32 (18.7)	139 (81.3)	Ref	Ref
Primary and below	29 (33.3)	56 (66.7)	0.46 (0.25-0.83)	1.00 (0.46-2.19)
**Family type**				
Nuclear	45 (28.0)	116 (72.0)	2.04 (1.06-3.91)	1.65 (1.21-2.01)
Joint/Extended	15 (16.0)	79 (84.0)	Ref	Ref
**Marriage type**				
Love	12 (21.1)	45 (78.9)	Ref	Ref
Arrange	36 (20.8)	137 (79.2)	1.01 (0.48-2.11)	0.94 (0.39-2.28)
Eloped	12 (48.0)	13 (52.0)	0.28 (0.10-0.79)	
**Duration of marriage (in years)**				
≤ 5	6 (18.8)	26 (81.3)	Ref	
6-10	10 (21.7)	36 (78.3)	0.83 (0.26-2.57)	
11-15	14 (20.9)	53 (79.1)	0.87 (0.30-2.53)	
More than 15	30 (27.3)	80 (72.7)	0.61 (0.23-1.64)	
**Occupation**				
Homemaker	19 (24.1)	60 (75.9)	Ref	Ref
Agriculture	25 (20.0)	100 (80.0)	1.26 (0.64-2.49)	1.06 (0.46-2.47)
Business	13 (44.8)	16 (55.2)	0.39 (0.15-0.95)	0.29 (0.13-1.5)
Others	3 (13.6)	19 (86.4)	2.00 (0.53-7.52)	1.59 (0.63-6.5)
**Number of children**				
1	20 (25.3)	59 (74.7)	Ref	
2	26 (20.8)	99 (79.2)	1.29 (0.66-2.51)	
3 and above	13 (34.2)	25 (65.8)	0.65 (0.28-1.51)	
**Income**				
51000 and above	19 (18.8)	82 (81.2)	Ref	
Below or equal to 50000	41 (26.6)	113 (73.4)	0.63 (0.34-1.18)	
**Family debt**				
Yes	48 (31.8)	103 (68.2)	3.57 (1.82-6.99)	2.23 (1.19-5.89)
No	12 (11.5)	92 (88.5)	Ref	Ref

*COR: Crude Odds Ratio, AOR: Adjusted Odds Ratio, Ref: Reference*

### Association between behavioral health-related characteristics and depression

Table 5 shows higher odds of depression among participants who had consumed alcohol (AOR: 1.76, 95% CI: 1.15 – 5.56, p = 0.041).

**Table 5:** Multivariate logistic regression analysis between behavioral health-related characteristics and depression.

Characteristics	Depression		Bivariate analysis	Multivariate logistic regression analysis
	Yes	No	COR (95% CI)	AOR (95% CI)
	n (%)	n (%)		
**Smoking/tobacco use**				
Yes	1 (50.0)	1 (50.0)	3.28 (0.78-4.16)	
No	59 (23.3)	194 (76.7)	Ref	
**Alcohol consumption**				
Yes	13 (52.0)	12 (48.0)	4.21 (1.39-9.16)	1.76 (1.15-5.56)
No	47 (20.0)	183 (80.0)	Ref	Ref
**Type of physical activity**				
Moderate intensity	4 (33.3)	8 (66.7)	Ref	
Light intensity	56 (23.0)	187 (77.0)	1.67 (0.48-5.75)	
**Health Illness within 6 months**				
Yes	17 (42.5)	23 (57.5)	3.01 (1.48-6.07)	Ref
No	43 (20.0)	172 (80.0)	Ref	0.66 (0.27-1.58)
**Mental illness**				
Yes	4 (66.7)	2 (33.3)	3.23 (0.78-16.76)	
No	13 (38.2)	21 (61.8)	Ref	

*COR: Crude Odds Ratio, AOR: Adjusted Odds Ratio, Ref: Reference*

### Association between autonomy and migration-related characteristics and depression

Table 6 shows that participants who reported not having emotional support from their family had significantly higher odds of depression compared to those who had emotional family support (AOR: 1.51, 95% CI: 1.12–1.96, p = 0.031).

**Table 6:** Multivariate logistic regression analysis of autonomy and migration-related characteristics, and depression.

Characteristics	Depression		Bivariate analysis	Multivariate logistic regression analysis
	Yes	No	COR (95% CI)	AOR (95% CI)
	n (%)	n (%)		
**Household head**				
Self	50 (24.4)	155 (75.6)	Ref	
Others	10 (20.0)	40 (80.0)	1.29 (0.60-2.76)	
**Decision on husband’s earning**				
Both (husband and self)	39 (23.8)	125 (76.2)	Ref	
Self	14 (26.4)	39 (40.5)	0.86 (0.42-1.76)	
Others	7 (18.4)	31 (81.6)	1.38 (0.56-3.38)	
**Decision on healthcare**				
Self	54 (24.7)	165 (75.3)	Ref	
Others	6 (16.7)	30 (83.3)	1.63 (0.64-4.14)	
**Decision on the purchase of goods**				
Self	54 (24.3)	168 (75.7)	Ref	
Others	6 (18.2)	27 (81.8)	1.44 (0.56-3.68)	
**Family support (Emotional)**				
Yes	49 (21.2)	182 (78.8)	Ref	Ref
No	11 (52.3)	10 (47.7)	0.24 (0.14-0.90)	1.51 (1.12-1.96)
**Family support (Physical)**				
Yes	45 (22.8)	152 (77.2)	Ref	
No	13 (23.6)	42 (76.4)	0.95 (0.47-1.93)	
**Social support (emotional)**				
Yes	29 (18.6)	127 (81.4)	Ref	Ref
No	26 (28.3)	66 (71.6)	0.58 (0.31-0.96)	1.03 (0.48-2.22)
**Social support (physical)**				
Yes	3 (11.1)	24 (88.9)	Ref	
No	52 (23.5)	169 (76.5)	0.40 (0.11-1.40)	
**Community group involvement**				
Yes	33 (21.6)	120 (78.4)	Ref	
No	27 (26.5)	75 (73.5)	0.76 (0.42-1.37)	
**Husband occupation**				
Labour	14 (25.5)	41 (74.5)	Ref	
Driver	14 (26.9)	38 (73.1)	0.92 (0.39-2.19)	
Security guard	5 (14.7)	29 (85.3)	1.98 (0.64-6.10)	
Hotel worker	15 (9.4)	25 (62.5)	0.56 (0.23-1.37)	
Others (technician, agriculture)	12 (16.2)	62 (83.8)	1.76 (0.74-4.19)	
**Frequency of remittance**				
Within a month	47 (23.7)	151 (76.3)	Ref	
More than a month	13 (22.8)	44 (77.2)	1.05 (0.52-2.12)	

*COR: Crude Odds Ratio, AOR: Adjusted Odds Ratio, Ref: Reference*

## Discussion

This study shows that nearly one-fourth (23.5%) of the wives of migrant workers had a prevalence of depression, which is almost similar to studies conducted in rural Mexico (23.2%) [24], Tajikistan (26%) [25] and in the North East part of Bangladesh (28.3%) [4]. However, this finding is lower than a study conducted in Chitwan, Nepal (79%) [10] but higher than in Nawalparasi, Nepal (6.5%) [9]. In comparison, a study conducted in Sri Lanka among left-behind families regarding common mental disorders depicted that 7.2% of married females had a prevalence of depression [5]. The variations in prevalence might be due to diverse contextual factors like socio-demographic factors, economic conditions, and cultural differences [26–28].

Brahmin/Chhetri ethnicity had higher odds of being depressed than others in this study. A secondary data analysis of a nationally representative survey of Nepal also showed that Janajati ethnic groups had less likelihood of being depressed compared to Brahmin/Chhetri [29]. This finding is in sharp contrast with the study conducted in Chitwan, Nepal, which shows that Janajati and other ethnic groups of married females had a 1.5 times higher risk of depression than Brahmin/Chhetri females [10]. Various cultural and social norms shape individual thought systems and influence mental health [30]. In this regard, Brahmins/Chhetri groups may have high pressure to uphold their existing cultural and social norms in the society, such as maintaining family prestige, fulfilling patriarchal expectations, etc., which may affect their psychological health.

Women who attended primary and below education were less likely to be depressed than those attending secondary/above levels of education. A similar finding was observed in a study conducted in Canada, where the lowest lifetime depression prevalence (9.1%) was seen among individuals who had attained below-secondary-level education [31]. This finding is in contrast with several studies conducted in Nepal [10], Sri Lanka [5], Bangladesh [4], and Mexico [24], which showed that women attending lower levels of education suffer more from depression. This might be due to inadequate employment opportunities and low income regardless of higher education, which leads to financial hardship in the family [32]. Married females may face academic stress due to time constraints as they have to manage their educational and household tasks in parallel, which may create mental health issues [33].

In this study, women belonging to nuclear families were 1.6 times more likely to be depressed than those living in non-nuclear families, which contrasts with the study conducted in Azad Jammu & Kashmir, Pakistan [17]. However, studies from Nepal [10] and India [12] reported no significant association between family type and depression. In nuclear families, females often face several challenges in decision-making regarding childcare, household matters, and experience limited social interaction with their husbands [12,34,35]. Additionally, they tend to shoulder multiple responsibilities, including household chores, caregiving, and childcare, without external support, which may lead to stress, burnout, and depression [17,36]. On the other hand, in joint or extended families, women may face strict societal expectations, conflicts with in-laws, disputes over financial decision-making with their husband’s earnings, limited autonomy, and inadequate emotional support within the family [10,15,19]. These situations might create feelings of isolation and further increase their vulnerability to depression among wives of migrant workers.

Women with arranged marriages were more likely to be depressed than those with love marriages, in this study. This is due to frequent spousal disagreement, which significantly increases depression among women [37]. Furthermore, individuals who performed arranged marriages tend to have interpersonal problems relating to domineering behavior and try to adhere more to social and family norms, which may cause a psychological impact [38].

Similarly, women with family debt had 2.2 times higher odds of being depressed than those with no family debt. This finding is supported by studies conducted in Sri Lanka [5] and the United States of America [39]. The main reason may be that migrant workers take out loans during the migratory process. Consequently, their wives are often responsible for repaying these debts, which may cause them to experience stress-related disorders and ultimately lead to depression [5].

Women with no or light-intensity physical exercise have a higher chance of depression compared to those who perform moderate-intensity physical activity, according to this study. This finding is supported by several studies done in Tasmania [40], Australia [41,42], and Europe [43]. Regular physical activity leads to better sleep patterns [44], which can mitigate depressive symptoms. Moreover, achieving fitness goals and improving physical health can boost self-esteem, countering feelings of worthlessness, which are often linked to depression [45]. Physiologically, these results may be due to increased serotonin level in the brain, which is associated with moods during physical activity [46]. Exercise also increases brain-derived neurotrophic factor, which supports brain health and reduces depression symptoms [47].

This study shows that women with existing health illnesses have higher odds of having depression compared to those without any illness. This finding is consistent with several studies conducted in Bangladesh [48], the United Kingdom [49] and India [50]. People with medical conditions are at higher risk of depression, which can worsen the outcomes of both conditions [50]. Physical illness increases the risk of depression in two ways: psychological factors (such as stress or challenges caused by the illness), and biological factors [51]. Depression is partly biological and linked to the illness itself and the body’s systems that affect depressive reactions [51].

## Strengths and limitations

This study addressed a significant research gap by focusing on the mental health status of left-behind wives of migrant workers, a population that has received limited attention in existing literature. The study used a validated tool to measure depression, enhancing the reliability and validity of the findings. As this study is conducted in community settings using multi-stage random sampling, the results are more generalizable in other similar community settings in resource-limited countries like Nepal.

This research, however, is subject to some limitations. The cross-sectional design limits the causal inferences about the relationship between depression and its associated factors. This study did not include wives of non-migrants, making it impossible to determine whether the observed associations are specific to migrant households. Therefore, the findings cannot be generalized to wives of non-migrants. Future research should adopt longitudinal designs with wider geographic representation and comparative analyses to build a more comprehensive understanding of the mental health impacts of the migration of husbands on left-behind spouses.

## Conclusion

Nearly one-fourth of the wives of migrant workers experienced depression. This study demonstrated that the likelihood of depression among wives of migrant workers was influenced by ethnic disparities, family types, marriage types, family debt, and health illness. This study highlights the need for targeted interventions to reduce depression among wives of migrant workers. Community-based mental health awareness programs, family-based counselling, and community-based financial initiative strategies can help address stress and strengthen coping skills. Integrating routine health screenings, strengthening peer support networks, and prioritizing migrant workers’ families in national mental health policies are essential to improve the well-being of this vulnerable group.

## Supporting information

S1 Dataset Data for analysis. (XLSX)
